# Evaluation of lymphocytes CD4+ and CD8+ and expression of ZAP-70 kinase on CD3+ and CD19+ lymphocytes in obese patients undergoing laparoscopic cholecystectomy

**DOI:** 10.1007/s00464-012-2527-6

**Published:** 2012-10-06

**Authors:** Kamil Torres, Anna Torres, Andrzej Chrościcki, Ryszard Maciejewski, Sebastian Radej, Jacek Roliński, Łukasz Pietrzyk, Grzegorz Wallner

**Affiliations:** 1 Laboratory of Biostructure, Human Anatomy Department, Medical University of Lublin, Jaczewskiego 4, 20-094 Lublin, Poland; 2 Department of General and Oncological Surgery, District Specialist Hospital, Lublin, Poland; 3 Department of Clinical Immunology, Medical University of Lublin, Lublin, Poland; 4 Department of General and Gastroenterological Surgery, and Surgical Oncology of the Alimentary Tract, Medical University of Lublin, Lublin, Poland

**Keywords:** Laparoscopy, Obesity, Pneumoperitoneum, T cells, ZAP-70 kinase, CD4+, CD8+

## Abstract

**Background:**

Obesity has become a global epidemic and a leading metabolic disease in the world. Laparoscopic surgeries may influence the function of the immunologic system. The percentages of CD4+ and CD8+ T lymphocyte cells have been described as prognostic factors for patients undergoing abdominal surgeries. This study aimed to evaluate the changes in CD4+ and CD8+ T lymphocyte cells, the ratio of CD4+ to CD8+ cells, and the ZAP-70 kinase expression on T CD3+ and B CD19+ cells in obese and normal-weight individuals undergoing laparoscopic cholecystectomy (LC).

**Methods:**

The study group consisted of 46 asymptomatic patients with gallstones shown by ultrasound examination but without signs of any gallbladder complications. The patients underwent planned LC. Blood samples were obtained at three times, and the percentages of studied cells were measured by flow cytometry. Patients were enrolled to two groups: N group (body mass index [BMI], ≤25 kg/m^2^) and O group (BMI, ≥30 kg/m^2^). For statistical analysis, the Mann–Whitney *U* test and the Wilcoxon matched-pairs signed-ranks test were used. All *p* values lower than 0.05 were considered significant.

**Results:**

The percentage of CD4+ T cells did not differ between the N and O groups before or after the surgery. Only in the N group did the percentage of CD4+ lymphocytes increase from 0 to 48 h. A higher percentage of CD8+ lymphocytes was observed in the O group postoperatively than in the N group. Differences of ZAP-70 kinase expression in the O group were observed at 24 and 48 h of the study. Decreased expression of ZAP-70 kinase was shown in the N group at both 0–24 and 24–48 h. In the O group, this tendency was noted at 24–48 h.

**Conclusions:**

Immunologic activation after LC was confirmed in both weight groups. However, higher modulation, more typical for open surgeries, was observed in the obese group.

According to the World Health Organization, obesity has become a global epidemic and a leading metabolic disease in the world [1, 2]. Obesity is associated with a higher incidence of cardiovascular diseases, metabolic disorders, and alterations in the immunologic profile [3]. The effect of carbon dioxide pneumoperitoneum used during minimally invasive surgeries on the immunologic function has not been fully explained, especially in obese individuals (BMI, >30 kg/m^2^) [4, 5].

Percentages of CD4+ and CD8+ T lymphocyte populations together with the CD4+ to CD8+ ratio were found to predict the outcome for patients undergoing abdominal surgeries [6]. In selective activation of lymphocytes, ZAP-70 kinase plays an important role and was detected on both lymphocytes T and B. As a cytoplasmic protein tyrosine, ZAP-70 kinase initiates a T cell response through the antigen receptor [7].

Decreased expression of ZAP-70 kinase was observed in patients with immunologic disorders and cancer, whereas high levels of ZAP-70 kinase expression were observed in B cells of patients with chronic lymphocytic leukemia [7]. An increased prevalence of cancer and worse postoperative progress were observed among obese individuals [8]. However, the influence of pneumoperitoneum on the changes in the immunologic system, including populations of CD4+ and CD8+ lymphocytes as well as ZAP-70 kinase expression in obese individuals, were not fully explained [4].

This study aimed to evaluate changes in CD4+ and CD8+ populations of T lymphocytes, the CD4+ to CD8+ ratio, and the expression of ZAP-70 kinase on T CD3+ and B CD19+ cells in obese and normal-weight individuals undergoing laparoscopic cholecystectomy.

## Materials and methods

The study investigated patients with cholelithiasis admitted to the General and Oncological Surgery Department of the District Specialist Hospital of Lublin, Poland. All the patients were informed about the aims of the study, and written consent was obtained from each patient. The study was approved by the Ethical Committee at the Medical University of Lublin (decision no. KE-0254/240/2008).

The study group consisted of 46 asymptomatic patients with gallstones shown by ultrasound examination but without signs of any gallbladder complications (e.g., empyema, hydrops, wall necrosis, jaundice, and others). The exclusion criteria specified symptoms of acute cholecystitis as well as a history of diabetes and immunologic disorders or allergies.

All the patients underwent laparoscopic cholecystectomy with standard values of carbon dioxide pneumoperitoneum (12–14 mmHg). The patients underwent surgery performed via a four-trocar technique. Anatomic recognition of Calot’s triangle was performed in each case. The cystic duct and cystic artery were clipped and cut. The gallbladder with Calot’s triangle lymph node was removed in a medical protector through the incision below the umbilicus. No complications during the postoperative period were reported for any of the patients. In each case, the gallbladder was evaluated by pathologic examination, and no malignancy was observed.

### Blood collection

Blood samples were obtained before the surgery as well as 24 and 48 h after the surgery.

### Flow cytometric evaluation

Whole blood samples (100 μL) and mononuclear cells (1 × 10^6^ cells/mL) from Calot’s triangle lymph node were stained with fluorochrome-conjugated antibodies anti-cell-surface markers and incubated for 15 min in darkness and room temperature. The cells were stained with the following antibody conjugates: anti-CD45 FITC/anti-CD14 PE, anti-CD4 FITC/anti-CD8 PE, and anti-CD3 FITC (Becton–Dickinson, San Jose, CA, USA).

### Negative control condition

The fluorescence-minus-one method or the unstained cell was used. At the end of the incubation, Cytofix/Cytoperm solution (Becton–Dickinson) was added to the samples, and incubation continued for next 15 min. The cells were washed and analyzed by flow cytometry on a FACSCalibur instrument (Becton–Dickinson). After fixing, the cells were stained with anti-CD3 FITC antibodies and pelleted.

### Intracellular ZAP-70 kinase detection

The BD Perm/Wash buffer (Becton–Dickinson) and anti-ZAP-70 PE (Becton–Dickinson) were added to the appropriate tubes and incubated for 15 min in darkness and room temperature. Finally, the cells were washed and analyzed by flow cytometry. For each analysis, 20,000 events were acquired and analyzed using CellQuest software (Becton Dickinson, Franklin Lakes, New Jersey, USA).

### Statistical analysis

The Mann–Whitney *U* test was used to assess differences between studied patient populations. Differences from baseline within each group were evaluated using the Wilcoxon matched-pairs signed-ranks test. All *p* values lower than 0.05 were considered significant. Statistical analysis was performed using 16.0 SPSS software (SPSS, Chicago, IL, USA).

## Results

The patients were assigned to one of two groups: group N (*n* = 23, BMI ≤25 kg/m^2^) and group O (*n* = 23, BMI ≥ 30 kg/m^2^). The median age of the patients in both groups was 55 years, ranging from 21 to 71 years in group N and from 27 to 76 years in group O. The ratio of female to male patients was 16:7 in both groups. The two groups differed significantly in terms of BMI (*p* < 0.001). The BMI was 22.65 kg/m^2^ (range, 18.29–24.98 kg/m^2^) in group N and 31.96 kg/m^2^ (range, 30.02–40.39 kg/m^2^) in group O.

The two groups did not differ significantly in duration of surgery. The median duration of surgery was 50.50 min in group N and 45.00 min in group O. Before the surgery, each patient was assessed according to the American Society of Anesthesiology (ASA) scale, and statistical analysis did not show any significant differences between the two groups. Additionally, no differences in biochemical (aspartate aminotransferase, alanine aminotransferase, creatinine) or hematologic (hemoglobin, hematocrit, platelets) Query parameters were observed in examinations before or after the surgery. The median body temperatures and white blood counts (WBC) were similar (normal ranges) in both groups during the postoperative period. No complications (e.g., fever or wound infections) were observed during the postoperative period in either study group.

**Fig. 1:**
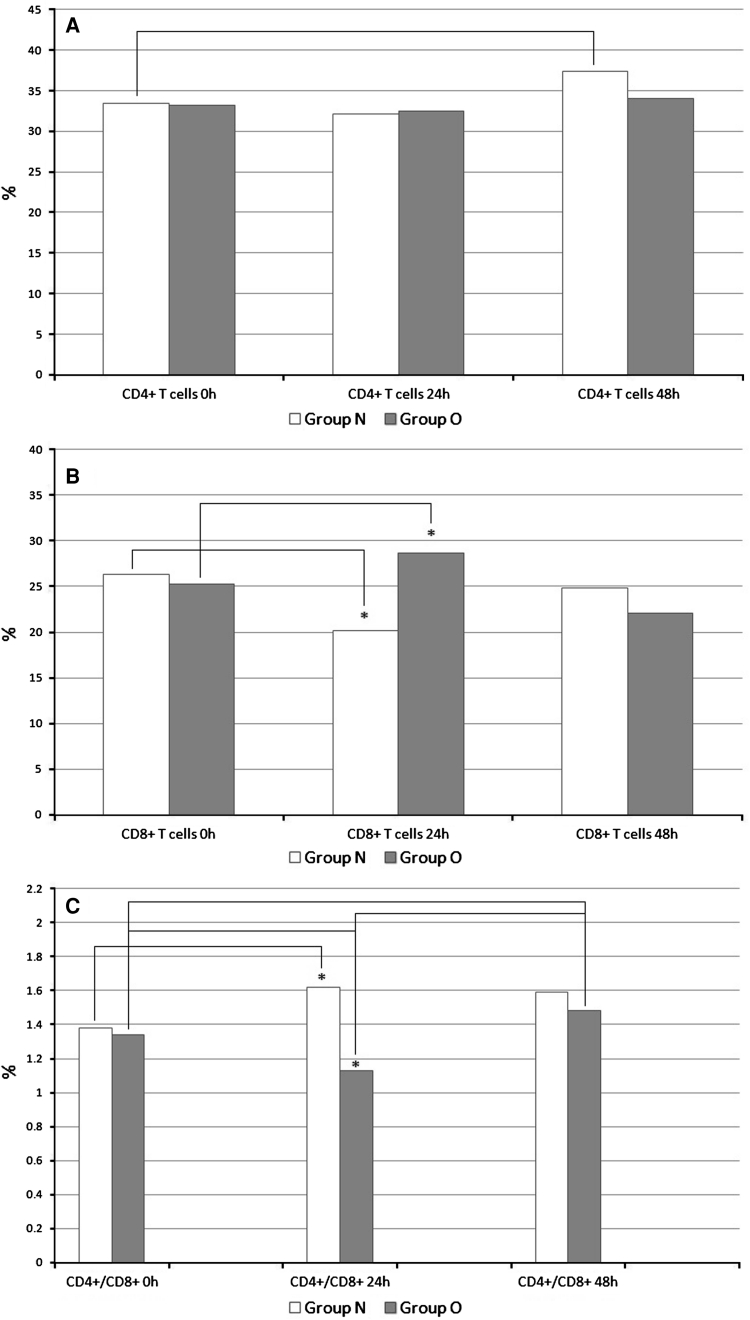
**a** Percentage of CD4+ T cells and its changes in three studied periods. *p < 0.05, group N versus group O; *p* < 0.05, postoperative percentages versus baseline percentages. **b** Percentage of CD8+ T cells and its changes in three studied periods. *p < 0.05, group N versus group O; *p* < 0.05, postoperative percentages versus baseline percentages. **c** Ratio of CD4+ to CD8+ (CD4+/CD8+) T cells and its changes in three studied periods. *p < 0.05, group N versus group O; <*p* < 0.05, postoperative percentages versus baseline percentages

No statistically significant differences in the percentage of CD4+ and CD8+ T cells, the ratio of CD4+/CD8+ lymphocytes, or the T CD3+ and B CD19+ cells with expression of ZAP-70 kinase were observed between the two groups before surgery. The percentages of CD4+ T cells did not differ 24 or 48 h after surgery between the two groups. However, in group N, the percentage of CD4+ T cells increased significantly between the preoperative period and 48 h after surgery. In the obese group, such a tendency was not observed. The percentage of CD4+ T cells and its changes in both groups are presented in Fig. 1A.

A higher percentage of CD8+ T cells was observed 24 h after surgery in the obese group than in the normal-weight group. No differences in percentage of CD8+ T cells were observed before and 48 h after the surgery between the groups. A significant increase in the percentage of CD8+ T cells was observed in obese group compared with the levels before surgery and 24 h after surgery. At the same time, a significantly decreased percentage of CD8+ T cells was observed in group N. Comparison of CD8+ T cell percentage values between postoperative hours 24 and 48 showed a significantly increased percentage in the normal-weight group and a decrease in the obese group (Fig. 1B).

The ratio of CD4+ to CD8+ lymphocytes was evaluated in the study groups before surgery and then 24 and 48 h afterward (Fig. 1C).We observed a significantly higher ratio of CD4+ to CD8+ lymphocyte cells in the normal-weight group 24 h after the surgery than in group O. The ratio of CD4+ to CD8+ T cells increased significantly in group N between the time before surgery and 24 h after the surgery compared with group O, which showed a significantly decreased ratio. In the obese group, the ratio of CD4+ to CD8+ T cells significantly increased between 24 and 48 h after the surgery.

**Fig. 2:**
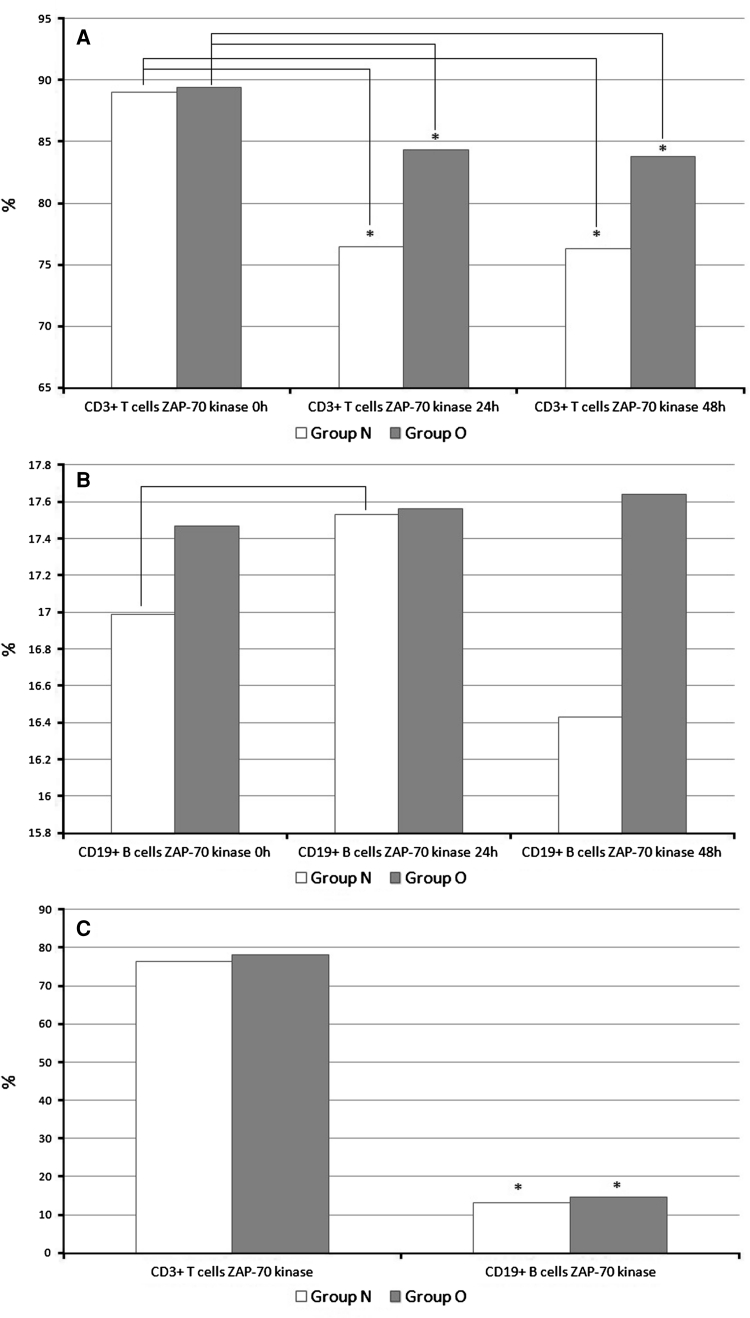
**a** Percentage of CD3+/ZAP-70 kinase T cells and its changes in three studied periods. *p < 0.05, group N versus group O; *p* < 0.05, postoperative percentages versus baseline percentages. **b** Percentage of CD19+/ZAP-70 kinase B cells and its changes in three studied periods; *p* < 0.05, postoperative percentages versus baseline percentages. **c** Percentage of CD3+/ZAP-70 kinase T cells and CD19+/ZAP-70 B cells in Calot’s triangle lymph node. *p < 0.05, group N versus group O

The percentage of CD3+/ZAP-70 kinase T cells did not differ between the groups before surgery. A significantly higher percentage of CD3+/ZAP-70 kinase T cells was observed at both 24 and 48 h after the surgery in group O compared with group N. However, in both groups, a significant decrease was observed between the surgery and 24 h afterward. The percentages of CD3+/Zap-70 kinase T cells and their changes are shown in Fig. 2A.

No differences in percentage of CD19+/ZAP-70 kinase B cells were observed between the groups during the postoperative period (Fig. 2B). However, the percentage of CD19+/ZAP-70 kinase B cells increased significantly before the surgery and 24 h afterward in group N compared with group O.

The ZAP-70 kinase expression on CD3+ T lymphocytes isolated from the Calot’s triangle lymphatic node showed no significant differences. However, a higher percentage of CD19+/ZAP-70 kinase B cells of Calot’s triangle lymphatic node were observed in group O. The results are presented in Fig. 2C.

## Discussion

Despite its low invasiveness, laparoscopic cholecystectomy impairs immunologic balance, especially in obese individuals [9]. Di Vita et al. [10] reported the association between increased intraabdominal pressure and immunologic alteration in obese patients. Excessive fat tissue often is increased due to excessive fat accumulation in obese individuals, causing increased intraabdominal pressure. Consequently, carbon dioxide pneumoperitoneum used during laparoscopic procedures for this group of patients influences the cardiopulmonary, nervous, and immunologic systems to a greater extent [8, 11]. In addition, immunologic changes in the fatty tissue of obese individuals also were observed in a number of studies [12, 13]. Taken together, these data suggest that the immunologic response to operative trauma connected with laparoscopic procedures could be different for patients with a highly increased BMI.

The response of the immunologic system of obese individuals to carbon dioxide pneumoperitoneum remains complex and unclear [14]. Findings show that CD4+ and CD8+ T cells play an important role in the immunologic response after surgery [14]. The maturation of CD4+ and CD8+ lymphocytes requires transduction of the T cell antigen receptor, which takes place in the thymus and involves tyrosine phosphorylation of the protein tyrosine kinase ZAP-70 by p56(lck) [15].

Findings have determined that ZAP-70 kinase is a key signal transduction molecule in T cells. Activated CD4+ T cells play a significant role in several autoimmunologic disorders such as rheumatoid arthritis and in the postoperative response [16]. Pelekanou et al. [17] observed a decrease of CD4+ T cells in patients with sepsis. However, the cellular and molecular mechanism of T cell activation in septic patients remains unclear [18].

In our study, the percentage of CD4+ T cells did not differ between the normal-weight (N) and obese (O) groups at any of the studied time points. However, only in the normal-weight group was the percentage of CD4+ lymphocyte cells increased within 48 h after the beginning of the surgery. No such phenomenon was observed in the obese group, which showed no significant change in the percentage of CD4+ cells.

Other authors have described similar changes as favorable prognostic factors and found them more characteristic of laparoscopic techniques than of open surgery [19]. Our study showed a higher percentage of CD8+ T cells in the obese group during the postoperative period. A similar cytotoxic immunologic response was observed by other authors in obese patients and patients undergoing conventional operations. Higher levels of CD8+ T lymphocytes suggest that the immunologic balance is impaired in favor of the cytotoxic cell-mediated response in the obese group [6].

Lausten et al. [19] observed a decrease in circulating CD4+ lymphocyte cells in patients who underwent surgery by conventional methods compared with those who had laparoscopic surgery. The data obtained in our study may suggest that the immunologic response of obese patients recovering from laparoscopic short-term procedures has a greater resemblance to the response of patients who underwent a conventional laparotomy. Similar observations were made by other authors for patients undergoing open cholecystectomy versus laparoscopic techniques [20]. Whitson et al. [21] observed a greater decrease in cytotoxic response in patients subjected to laparoscopic techniques than in those who underwent laparotomy. In the study performed by Bolla and Tuzzato [14], greater impairment of cell-mediated immune function after surgery was characteristic of patients who had surgery via the open technique compared with those subjected to laparoscopic techniques.

Our study showed no differences in ZAP-70 kinase expression between the study groups during the preoperative period. Such observation might suggest that the metabolic and immunologic activity of the fatty tissue does not impair the expression of ZAP-70 kinase in obese individuals and that the activation of T cells observed by other authors might therefore occur through a different pathway [2].

Our study showed, for the first time, differences of ZAP-70 kinase expression in the obese patients at both the 24th and 48th hour of the study compared with the normal-weight group. Decreased expression of ZAP-70 kinase was present in group N both between 0 and 24 h and between 24 and 48 h after surgery. This observation suggests a decreased immune activation compared with the obese group. In the obese group, a similar tendency was observed between 24 and 48 h after surgery.

The aforementioned data imply that the expression of ZAP-70 kinase is delayed and altered in the obese group. Such alteration might influence the activation of T lymphocytes in the early postoperative period and could explain the decreased levels of CD4+ T cells observed in obese individuals undergoing surgical procedures.

To evaluate the association between the local inflammatory status and the systemic expression of ZAP-70 kinase, the lymph node from Calot’s triangle was obtained and evaluated. The expression of ZAP-70 kinase on CD3+ T and CD19+ B cells was assessed. We did not find any differences in the inflammatory status between the study groups.

Injury and surgical trauma stimulate the regulation of CD4+ lymphocyte cells, and T cell receptors connected with ZAP-70 kinase are involved in this process. Upregulation of ZAP-70 kinase has been observed in the early activation of T cell receptors after burn injury [18]. Negishi et al. [22] showed that T cell development in ZAP-70 kinase-deficient mice was arrested at the transition from the CD4+ and CD8+ cells. Hanschen et al. [23] demonstrated the potential use of the phosphor-flow cytometer to evaluate the expression and upregulation of ZAP-70 kinase on T cells.

To date, altered expression of ZAP-70 kinase expression has been described in severe combined immunodeficiency disease (SCID) and chronic lymphocytic leukemia (B-CLL). Additionally, in both diseases, ZAP-70 kinase was considered a prognostic factor [7]. In obese individuals, activation of T cells might be induced by chronic low-grade inflammation, which is present in the fatty tissue and could be responsible for alterations of the immunologic response [24]. Arpaia et al. [25] observed that ZAP-70 kinase-deficient patients had no functional T cells. The proper activation of T cells might be essential for the proper wound healing and recovery after surgery [5].

Laparoscopic operations better preserve the distribution of T lymphocytes and maintain an elevated ratio of CD4+ to CD8+ lymphocyte cells, which is considered a prognostic factor [26]. In our study we confirmed the alteration of ZAP-70 kinase expression in obese patients after surgery, which could in turn influence levels of CD4+ lymphocytes and decrease the CD4+ to CD8+ (lymphocyte cells) ratio in favor of the cytotoxic component. The obtained results might be related to the short duration of the laparoscopic surgery and the lower activation of the immunologic system. However, no studies have described the expression of ZAP-70 kinase in obese patients undergoing laparoscopic surgeries. Further studies are required for a full explanation of these preliminary observations.

Although several studies have described immunologic changes after laparoscopic surgeries, our study did not show any correlations between immunologic alterations and complications during the early postoperative period. This could be due to the fact that the study was performed in a low-morbidity population of surgical patients and the operative times were short. However, we still could detect some differences in the studied immunologic parameters between obese and normal-weight individuals. The result of our study suggested that a potential to proper immunologic response induced by laparoscopic surgical trauma was better preserved in nonobese patients. The reason for this cannot be fully explained currently and could be connected with both endogenous and exogenous factors.

Nevertheless, our observations seem to have clinically important implications regarding the use of laparoscopic technique for obese patients. Increased abdominal pressure induced by carbon dioxide pneumoperitoneum is considered one of the negative factors connected with laparoscopy. Therefore, low-pressure pneumoperitoneum technique could be more beneficial for obese patients.

## Conclusions

Immune disturbances after laparoscopic cholecystectomy were observed both in patients with normal BMI and in obese patients. However, a higher modulation, more typical for open surgeries, was observed in the obese group.
